# Aberrant Pulmonary Vein Draining to Left Atrial Roof in a Patient Undergoing Percutaneous Circumferential Pulmonary Vein Isolation

**DOI:** 10.5812/cardiovascmed.13546

**Published:** 2013-10-28

**Authors:** Neshat Nazari, Hamidreza Pouraliakabr, Majid Haghjoo

**Affiliations:** 1 Cardiac Electrophysiology Research Center, Rajaie Cardiovascular Medical and Research Center, Iran University of Medical Sciences, Tehran, IR Iran; 2 Rajaie Cardiovascular Medical and Research Center, Iran University of Medical Sciences, Tehran, IR Iran

**Keywords:** Pulmonary Vein, Atrial Fibrillation, Aberrancy

**Figure 1.**
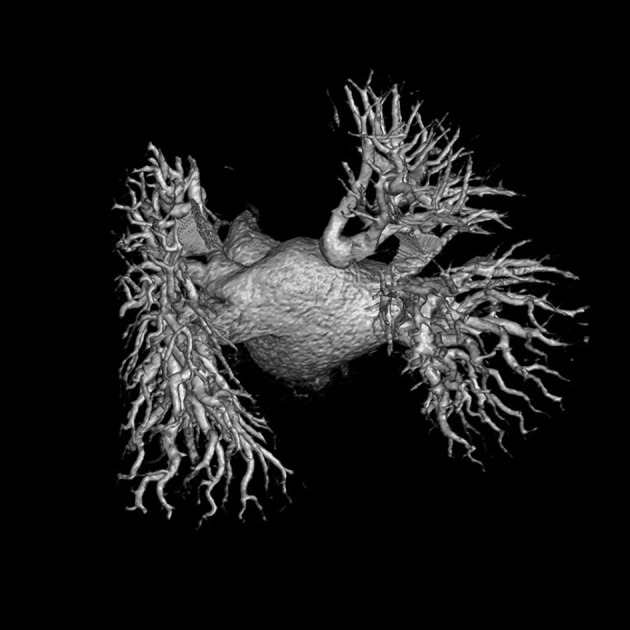
Aberrant Large Pulmonary Vein Draining into Mid-Part of Left Atrial Roof

The patient was a 61-year-old woman with frequent drug-refractory paroxysmal atrial fibrillation (AF). She had history of the systemic hypertension with normal coronary arteriogram. Pre-ablation transesophageal echo showed mild to moderate RV enlargement and medium-sized atrial septal defect (ASD). Multidetector computed tomography (MDCT) showed two left-sided pulmonary veins (PV), three right-sided PVs, and one aberrant large PV draining into mid-LA roof (Figure 1). This aberrant PV was draining posterior segment of the right upper lobe. During ablation, it was noted that ectopies from the aberrant PV frequently initiated AF. ASD was closed successfully using Amplatzer septal occluder. During follow-up, patient had no recurrence of AF. This abnormality was reported in 0.4% of the patients investigated with MDCT( 1 ) The possibility of this anomaly should be kept in mind to increase the ablation success and prevent complications such as cardiac tamponade.
